# Cerebellar damage with inflammation upregulates oxytocin receptor expression in Bergmann Glia

**DOI:** 10.1186/s13041-024-01114-5

**Published:** 2024-06-28

**Authors:** Ayumu Inutsuka, Aisa Hattori, Masahide Yoshida, Yuki Takayanagi, Tatsushi Onaka

**Affiliations:** Department of Physiology, Jichi Medical University, Shimotsuke, 323-0498 Japan

**Keywords:** Cerebellum, Oxytocin, Inflammation, Purkinje cell, Bergmann glia

## Abstract

The cerebellum plays an important role in cognitive and social functioning. Childhood damage in the cerebellum increases the risk of autism spectrum disorder. Cerebellar inflammation induces social avoidance in mice. Oxytocin regulates social relationship and expression pattern of the oxytocin receptor in the brain is related to social behaviors. However, the expression patterns of the oxytocin receptor in the cerebellum remain controversial. Here, we report that the expression patterns of the oxytocin receptor in the cerebellum are highly variable among knock-in transgenic lines. We used *Oxtr-Cre* knock-in mice combined with a fluorescent reporter line and found that oxytocin receptor expression in Bergmann glia was more variable than that in Purkinje cells. We found that physical damage with inflammation induced the selective upregulation of the oxytocin receptor in Bergmann glia. Our findings indicate high variability in oxytocin receptor expression in the cerebellum and suggest that the oxytocin receptor can affect neural processing in pathological conditions, such as inflammation.

**Supplementary Information:**

The online version contains supplementary material available at 10.1186/s13041-024-01114-5.

## Introduction

The cerebellum regulates body movements and cognitive functions. Cerebellar injury at birth increases the risk of autism spectrum disorder [1]. Purkinje cells (PCs) in the cerebellum receive multiple inputs from parallel fibers and project to the deep cerebellar nuclei. The cerebellar cerebrocortical circuit controls social behavior [2]. Another essential component of the cerebellum is the Bergmann glial cells (BGs) which are specialized astrocytes that wrap around the dendrites of PCs [3]. BGs modulate the firing patterns of PCs, and their modulation by G protein-coupled receptor (GPCR) can affect social behavior [4].

Oxytocin is a neuropeptide that affects multiple physiological responses, including social behaviors [5]. Although oxytocin is produced by oxytocin neurons in the hypothalamus, the oxytocin receptor (OXTR) is widely expressed in the brain, including in the cerebellum. The expression pattern of OXTR is associated with social behavior [6]. Although some reports suggest that PCs express OXTR [7], others suggest that BGs, and not PCs, express OXTR [8]. These contradictory findings have not been explained in detail.

In this study, we found large variations in the expression patterns of OXTR in the cerebellum among the transgenic lines, even though they were all knock-in mice. We also observed changes in the expression of OXTR in BGs during aging. Finally, we found that physical damage with inflammation selectively activated OXTR expression in BGs.

## Results

**Fig. 1:**
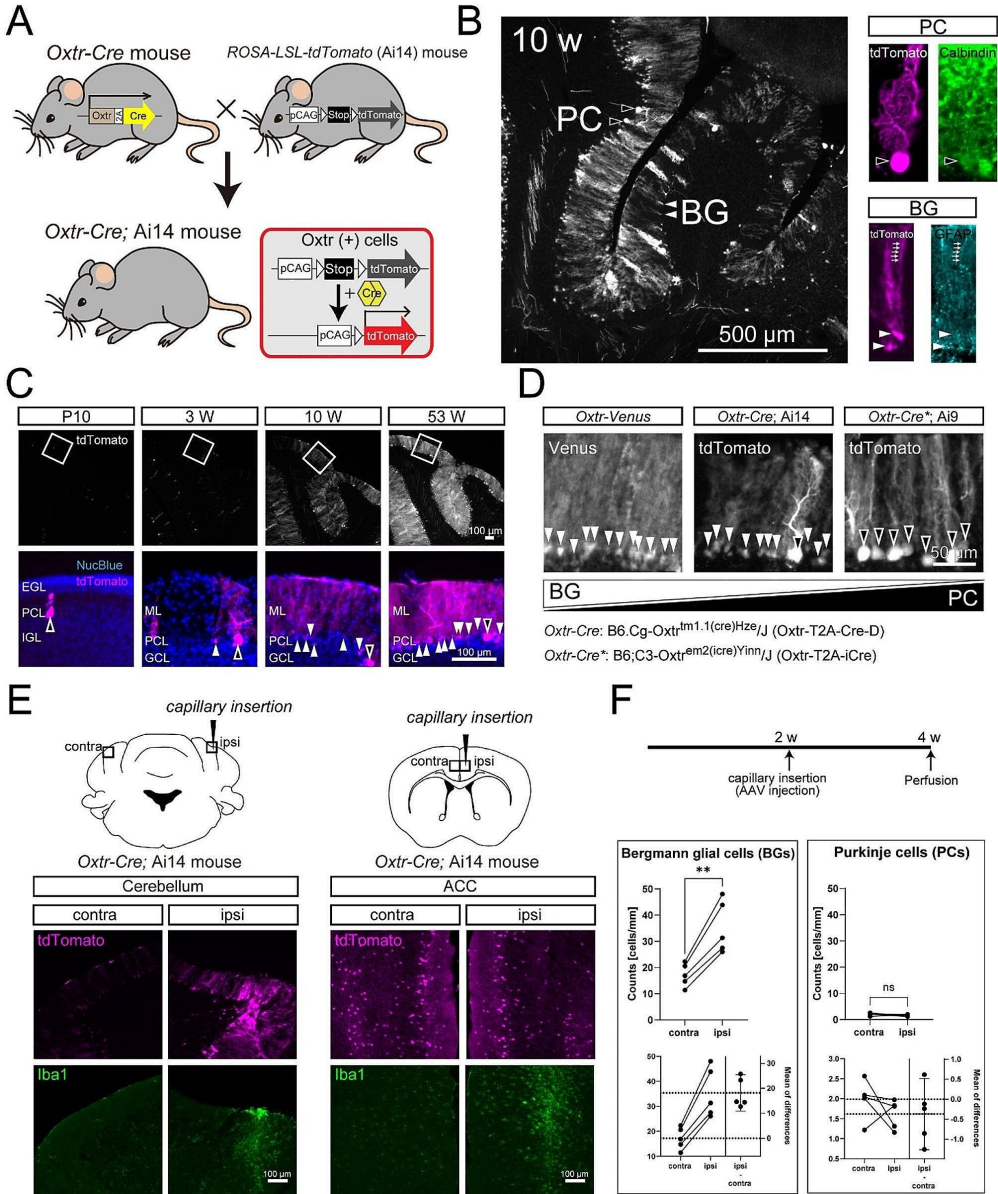
**A**, Diagram showing the experimental design of double-transgenic mice to observe OXTR expression. In *Oxtr-Cre*; ROSA-LSL-tdTomato (Ai14) mice, OXTR-expressing cells are visualized as tdTomato-expressing cells. **B**, A typical image of cerebellum slices showing tdTomato-positive cells in *Oxtr-Cre*; Ai14 mice. PCs, BGs, and other cells such as granule cells were observed. In the Purkinje cell layer, PCs were confirmed by calbindin immunostaining, while BGs were confirmed by GFAP immunostaining. Black arrowhead; a cell body of a PC, white arrowheads; cell bodies of BGs, white arrows; radial fibers of BGs. Scale bar = 500 μm. PC, Purkinje cell; BG, Bergmann glial cell. **C**, Developmental changes in expression patterns of OXTR in the cerebellum. Scale bar = 100 μm. IGL, internal granular cell layer; PCL, Purkinje cell layer; EGL, external granular cell layer; ML, molecular layer; GCL, granule cell layer. In the lower row, signal intensities of tdTomato were individually adjusted for clear visualization of cell morphology. We observed at least 3 mice at each developmental stage except 53 weeks (two mice). Black arrowheads; cell bodies of PCs, white arrowheads; cell bodies of BGs. **D**, Typical expression pattern of OXTR in the cerebellar Purkinje cell layer in three transgenic lines. Scale bar = 50 μm. Black arrowheads; cell bodies of PCs, white arrowheads; cell bodies of BGs. We observed at least 3 mice of each mouse line except Oxtr-Cre*; Ai9 (two mice). **E**, Selective local up-regulation of OXTR in the cerebellum at the site of capillary insertion. Scale bar = 100 μm. **F**, Quantitative analysis of tdTomato-positive cells on the ipsilateral and contralateral side of the cerebellum. ** *P* < 0.01 (*n* = 5)

To visualize OXTR expression patterns, we used an *Oxtr-Cre* knock-in mouse line combined with a reporter line. In double-transgenic *Oxtr-Cre*; *Rosa-LSL-tdTomato* (Ai14) mice, OXTR expression was detected as tdTomato expression (Fig. 1A). Brain slices of *Oxtr-Cre*; Ai14 mice showed tdTomato expression in both PCs and BGs of the cerebellum (Fig. 1B). PCs and BGs were identified by immunostaining with anti-calbindin and anti-glial fibrillary acidic protein (GFAP) antibodies as molecular markers respectively. Developmental changes in OXTR expression were investigated on postnatal days 10, 3 weeks, 10 weeks, and 53 weeks old (Fig. 1C). We observed a gradual increase in the number of tdTomato-positive cells in the Purkinje cell layer (PCL) of the cerebellum. The increase in the number of tdTomato-expressing cells in the PCL was mainly due to an increase in tdTomato-expressing BGs. Next, we compared the three transgenic lines that visualize OXTR-expressing cells. *Oxtr-Venus* knock-in mice showed an almost exclusive expression pattern in the BGs. We also examined mice created by crossbreeding another line of *Oxtr-Cre* knock-in mice [9] with *Rosa-LSL-tdTomato* (Ai9) reporter mice (described in the Methods section of the Supplemental Information). The mice showed tdTomato expression mainly in PCs. Therefore, the three transgenic lines exhibited different cerebellar OXTR expression patterns (Fig. 1D). Finally, we investigated the effects of the physical injury caused by glass capillary insertion into the cerebellum. We found that capillary insertion induced local upregulation of tdTomato in the cerebellum (Fig. 1E). Immunostaining of microglia using an anti-Iba1 antibody showed a local increase in reactive microglia on the ipsilateral side of the cerebellum compared with the contralateral side. We performed similar experiments in other brain areas, such as the anterior cingulate cortex (ACC), to examine whether OXTR upregulation was selective for BGs. We found no similar upregulation of tdTomato expression in these brain areas (Fig. 1E). Quantitative cell counting analysis indicated that this upregulation of OXTR in the cerebellum is a BG-specific phenomenon and that the number of tdTomato-expressing PCs was not affected by inflammation (Fig. 1F). We observed similar upregulation of OXTR one week after adeno-associated virus (AAV) injection, while we observed weaker upregulation two days after injection (Figure S1). Capillary insertion induced a local increase of Venus signal in the injured area of the cerebellum in *Oxtr-Venus* mice (Figure S2). Lipopolysaccharide (LPS) injection resulted in widespread upregulation of OXTR (Figure S3). We confirmed that there were no tdTomato-expressing cells in both the contralateral and ipsilateral sides of the insertion area in the cerebella of Cre-negative Ai14 mice (Figure S4). In the injured area of the cerebellum, we also observed local GFAP upregulation (Figure S5).

## Discussion

In this study, we demonstrated the high variability of OXTR expression in the cerebellum. OXTR expression in BGs was activated during development (Fig. 1C). It has not been confirmed that all tdTomato-expressing cells in *Oxtr-Cre*; Ai14 double-transgenic mice express OXTR at the time of fixation. Transient activation of the *Oxtr* gene can be observed as tdTomato-positive cells in this line. Therefore, cumulative weak activation may be observed as an increase in the number of tdTomato-positive cells. Nevertheless, these results suggest that physiological events can induce remarkable upregulation of OXTR in BGs that is not observed in other cells, such as PCs or astrocytes in the ACC.

The three transgenic lines showed highly variable expression patterns of OXTR in the cerebellum although they were all knock-in mice (Fig. 1D). These results clearly demonstrate that the selection of animal lines is important for investigating the physiological functions of OXTR in the cerebellum. Although transient expression of *Oxtr* in PCs during early development partially explains this difference, we cannot fully explain the mechanism at present. This topic should be addressed in future studies.

Differences in OXTR expression patterns in the forebrain can induce prominent changes in social behavior, from monogamous to promiscuous pair bonding [6]. The transcriptional regulation of *Oxtr* is highly variable among multiple bacterial artificial chromosome (BAC) transgenic lines with the same transgenes inserted into different genetic loci [10]. Our results show that this high transcriptional lability of *Oxtr* can be observed even in knock-in mice and provides an additional element: inflammation. We found that physical damage with inflammation in the cerebellum induced the specific activation of OXTR expression in BGs (Fig. 1E, F, S1, and S2). The glass capillary insertion in this study is a common protocol for local AAV brain injection [11]. Note that the negative control experiments with Cre-negative Ai14 mice clearly dispel the possibility of Cre-independent “leak” expression in Ai14 mice (Figure S4). BGs are generated selectively during the short period of E13.5-E14.5 [12]; therefore, it is not reasonable to consider the cell proliferation of tdTomato-expressing BGs in damaged areas.

It was reported that OXTR is not involved in the electrical properties of PCs and does not affect social behaviors, such as social interaction tests [7]. However, our results suggest that OXTR may mediate cell signaling under pathological conditions such as cerebellar infection. It has been reported that the insertion of an AAV-injecting needle induces inflammation and switches the genetic expression of neuron-specific enolase from PCs to BGs, and that LPS enhances this genetic regulation in BGs [13]. It was also reported that inflammation by LPS increased the OXTR expression via nuclear factor kappa B (NF-κΒ) in macrophages [14], and the promoter region of *Oxtr* includes several binding sites for NF-κΒ and interleukins [15]. In accordance with this report, we observed widespread upregulation of OXTR following LPS injection (Figure S3) and local GFAP upregulation in the injured brain area (Figure S5). Considering that cerebellar inflammation induces depression-like behaviors and social avoidance by changing the neural processing between the deep cerebellar nuclei and prefrontal cortex [16], our results indicate the need to investigate the physiological roles of OXTR in the cerebellum under pathological conditions.

### Electronic supplementary material

Below is the link to the electronic supplementary material.

Supplementary Material 1

Supplementary Material 2

Supplementary Material 3

Supplementary Material 4

Supplementary Material 5

Supplementary Material 6

## Data Availability

The experimental data that support the findings of this study are available in Figshare with the identifier 10.6084/m9.figshare.25309378.v1.

## Abbreviations

AAV: Adeno-associated virus
ACC: Anterior cingulate cortex
BAC: Bacterial artificial chromosome
BG: Bergmann glial cell
EGL: External granular cell layer
GCL: Granule cell layer
GFAP: Glial fibrillary acidic protein
GFP: Green fluorescent protein
GPCR: G protein-coupled receptor
H2B: Histone H2B
IGL: Internal granular cell layer
LPS: Lipopolysaccharide
ML: Molecular layer
NF-κB: Nuclear factor kappa B
OXTR: Oxytocin receptor
PC: Purkinje cell
PCL: Purkinje cell layer

## References

[1] Wang SS, Kloth AD, Badura A (2014). The cerebellum, sensitive periods, and autism. Neuron.

[2] Kelly E, Meng F, Fujita H, Morgado F, Kazemi Y, Rice LC, Ren C, Escamilla CO, Gibson JM, Sajadi S, Pendry RJ, Tan T, Ellegood J, Basson MA, Blakely RD, Dindot SV, Golzio C, Hahn MK, Katsanis N, Robins DM, Silverman JL, Singh KK, Wevrick R, Taylor MJ, Hammill C, Anagnostou E, Pfeiffer BE, Stoodley CJ, Lerch JP, du Lac S, Tsai PT (2020). Regulation of autism-relevant behaviors by cerebellar-prefrontal cortical circuits. Nat Neurosci.

[3] Ben Haim L, Rowitch DH (2017). Functional diversity of astrocytes in neural circuit regulation. Nat Rev Neurosci.

[4] Li C, Saliba NB, Martin H, Losurdo NA, Kolahdouzan K, Siddiqui R, Medeiros D, Li W (2023). Purkinje cell dopaminergic inputs to astrocytes regulate cerebellar-dependent behavior. Nat Commun.

[5] Takayanagi Y, Yoshida M, Bielsky IF, Ross HE, Kawamata M, Onaka T, Yanagisawa T, Kimura T, Matzuk MM, Young LJ, Nishimori K (2005). Pervasive social deficits, but normal parturition, in oxytocin receptor-deficient mice. Proc Natl Acad Sci U S A.

[6] Young LJ, Wang Z (2004). The neurobiology of pair bonding. Nat Neurosci.

[7] Shen LP, Li W, Pei LZ, Yin J, Xie ST, Li HZ, Yan C, Wang JJ, Zhang Q, Zhang XY, Zhu JN (2023). Oxytocin receptor in cerebellar Purkinje cells does not engage in autism-related behaviors. Cerebellum.

[8] Yoshida M, Takayanagi Y, Inoue K, Kimura T, Young LJ, Onaka T, Nishimori K (2009). Evidence that oxytocin exerts anxiolytic effects via oxytocin receptor expressed in serotonergic neurons in mice. J Neurosci.

[9] Inoue Y.U., Miwa H, Hori K, Kaneko R, Morimoto Y, Koike E, Asami J, Kamijo S, Yamada M, Hoshino M, Inoue T. Targeting neurons with functional oxytocin receptors: a Novel Set of simple Knock-In mouse lines for oxytocin receptor visualization and manipulation. eNeuro. 2022;9. 10.1523/ENEURO.0423-21.2022. 10.1523/ENEURO.0423-21.2022 PMC8856715 35082173

[10] Zhang Q, Nagai LAE, Tsukamoto M, Kandasamy LC, Inoue K, Pires MF, Shin M, Nagasawa Y, Sambuu T, Ogawa S, Nakai K, Itohara S, Young LJ (2023). Distal regulatory sequences contribute to diversity in brain oxytocin receptor expression patterns and social behavior. bioRxiv.

[11] Inutsuka A, Maejima S, Mizoguchi H, Kaneko R, Nomura R, Takanami K, Sakamoto H, Onaka T (2022). Nanobody-based RFP-dependent cre recombinase for selective anterograde tracing in RFP-expressing transgenic animals. Commun Biol.

[12] Sudarov A, Turnbull RK, Kim EJ, Lebel-Potter M, Guillemot F, Joyner AL (2011). Ascl1 genetics reveals insights into cerebellum local circuit assembly. J Neurosci.

[13] Sawada Y, Konno A, Nagaoka J, Hirai H (2016). Inflammation-induced reversible switch of the neuron-specific enolase promoter from Purkinje neurons to Bergmann glia. Sci Rep.

[14] Szeto A, Sun-Suslow N, Mendez AJ, Hernandez RI, Wagner KV, McCabe PM (2017). Regulation of the macrophage oxytocin receptor in response to inflammation. Am J Physiol Endocrinol Metab.

[15] Schmid B, Wong S, Mitchell BF (2001). Transcriptional regulation of oxytocin receptor by interleukin-1beta and interleukin-6. Endocrinology.

[16] Yamamoto M, Kim M, Imai H, Itakura Y, Ohtsuki G (2019). Microglia-Triggered plasticity of intrinsic excitability modulates psychomotor behaviors in Acute cerebellar inflammation. Cell Rep.

[17] Daigle TL, Madisen L, Hage TA, Valley MT, Knoblich U, Larsen RS, Takeno MM, Huang L, Gu H, Larsen R, Mills M, Bosma-Moody A, Siverts LA, Walker M, Graybuck LT, Yao Z, Fong O, Nguyen TN, Garren E, Lenz GH, Chavarha M, Pendergraft J, Harrington J, Hirokawa KE, Harris JA, Nicovich PR, McGraw MJ, Ollerenshaw DR, Smith KA, Baker CA, Ting JT, Sunkin SM, Lecoq J, Lin MZ, Boyden ES, Murphy GJ, da Costa NM, Waters J, Li L, Tasic B, Zeng H. A Suite of Transgenic Driver and Reporter Mouse Lines with Enhanced Brain-Cell-Type Targeting and Functionality, Cell 174 (2018) 465–480 e422. 10.1016/j.cell.2018.06.035. 10.1016/j.cell.2018.06.035 PMC6086366 30007418

