# Supplementary material for: Cerebellar damage with inflammation upregulates oxytocin receptor expression in Bergmann Glia
Source: Mol Brain. 2024 Jun 28;17:41. doi: 10.1186/s13041-024-01114-5 (PMC11214225; doi:10.1186/s13041-024-01114-5)
Supplement: Supplementary file 1 — Supplementary Material 1 [file 13041_2024_1114_MOESM1_ESM.docx]

**Supplementary Information**

**Materials and Methods**

**Animals**

All experimental procedures involving mice were approved by the Institutional Animal Experiment Committee of Jichi Medical University. The mice were maintained under a 12-hour light/dark cycle (light period: 7:30-19:30, dark period: 19:30 - 7:30) in a room with controlled temperature (22 ± 2°C) and humidity (55 ± 15%). Food and water were provided ad libitum. *Oxtr-Venus* (MGI:3838764) mice were first reported by (Yoshida, 2009) [8]. *Oxtr-T2A-Cre-D* (*Oxtr-Cre*) (RRID: IMSR_JAX:031303 [17]), Ai9 (RRID: IMSR_JAX:007909), and Ai14 (RRID: IMSR_JAX:0079114) transgenic mice were obtained from Jackson Laboratory. *Oxtr-T2A-iCre* (*Oxtr-Cre**) transgenic mice were generated and deposited to Jackson Laboratory (RRID: IMSR_JAX:037578) and RIKEN (RBRC11687) by Dr. Yukiko U. Inoue and their validation was published [9]. Brain samples of *Oxtr-T2A-iCre*; Ai9 were kindly gifted from Dr. Yukiko U. Inoue. In the left panel of **Figure 1B**, 10-week-old male *Oxtr-T2A-Cre-D*; Ai14 double-transgenic mice were used. For a clear visualization of cell morphology in sparse tdTomato-expressing cells, we used 4-week-old mice in **Figure 1B** (right panels). In **Figure 1C**, P10 and, 3-, 10-, and 53-week-old female *Oxtr-T2A-Cre-D*; Ai14 double-transgenic mice were used. In **Figure 1D**, 16-week-old male *Oxtr-Venus*, *Oxtr-T2A-iCre*; Ai9 double-transgenic mice, and *Oxtr-T2A-Cre-D*; Ai14 double-transgenic mice were used. In **Figure 1E**, 8-week-old male *Oxtr-T2A-Cre-D*; Ai14 double-transgenic mice were used. In **Figure 1F**, 4-week-old female *Oxtr-T2A-Cre-D*; Ai14 double-transgenic mice were used. In **Figures S1 and S3**, 4-week-old female *Oxtr-T2A-Cre-D*; Ai14 double-transgenic mice were used. In **Figure S2**, 4-week-old female *Oxtr-Venus* mice were used. In **Figure S4**, 11-week-old female Ai14 mice were used. In **Figure S5**, 4-week-old male *Oxtr-T2A-Cre-D*; Ai14 double-transgenic mice were used.

**Stereotaxic insertion of glass capillaries**

Glass capillaries (GC-1.5: Narishige, Tokyo, Japan) were pulled by a puller (PC-100: Narishige) and their tip diameter is approximately 100 μm. Surgeries for glass capillary insertion were performed using a stereotaxic instrument. Mice were anesthetized by intraperitoneal injection of Avertin. In experiments in **Figure 1E and S5**, A glass capillary was injected on the right side of the cerebellum (from bregma -5.8 mm, lateral 2.5 mm, ventral -1.0 mm) and in the right side of the anterior cingulate cortex (from bregma +1.0 mm, lateral 0.4 mm, ventral -1.3 mm) of 7-week-old male double-transgenic mice. The skull was drilled using a 2 mm diameter drill tip. The mice were perfused 1 week after injection. In experiments in **Figure 1F and S1**, A glass capillary was injected in the right crus of the cerebellum (from lambda -2.6 mm, lateral 2.1 mm, ventral -1.0 mm) of female double-transgenic mice and 1.2 μL of AAV-CAG-H2B-GFP-WPRE (2 × 10 ^12^ vg/mL) was injected for visualizing the insertion site. H2B, histone H2B; GFP, green fluorescent protein. The mice were perfused two days, one week and two weeks after injection at 4-week-old. In experiments in **Figure S2**, A glass capillary was injected in the right crus of the cerebellum (from lambda -2.6 mm, lateral 2.1 mm, ventral -1.0 mm) of 4-week-old female transgenic mice and 1.2 μL of AAV-CAG-H2B-mRFP1-WPRE (1 × 10^12^ vg/mL) was injected for visualizing the insertion site. The mice were perfused 2 days after injection. In experiments in **Figure S3**, a glass capillary was inserted in the right crus of the cerebellum (from lambda -2.6 mm, lateral 2.1 mm, ventral -1.0 mm) of 3-week-old female double-transgenic mice and 5 uL of LPS (1mg/mL in artificial cerebrospinal fluid [aCSF]) or 1.2 μL of AAV-CAG-H2B-GFP-WPRE (2 × 10^12^ vg/mL) was injected for visualizing the insertion site. The mice were perfused one week after injection. In experiments in **Figure S4**, a glass capillary was inserted in the right crus of the cerebellum (from lambda -2.6 mm, lateral 2.1 mm, ventral -1.0 mm) of 10-week-old female transgenic mice and 1.2 μL of AAV-CAG-H2B-GFP-WPRE (2 × 10^12^ vg/mL) was injected to visualize the insertion site. The mice were perfused one week after injection.

**Virus**

All AAV vectors were produced using the AAV Helper-Free System (Agilent Technologies, Santa Clara, CA) and purified using previously published methods [11]. Briefly, HEK293 cells were transfected with a pAAV vector plasmid that included the genes of interest, pHelper, and pAAV-RC (PHP.eb), using the standard calcium phosphate method. Three days later, the transfected cells were collected and suspended in artificial cerebrospinal fluid (aCSF; 124 mM NaCl, 3 mM KCl, 26 mM NaHCO_3_, 2 mM CaCl_2_, 1 mM MgSO4, 1.25 mM KH_2_PO_4_, and 10 mM D-glucose). After four freeze-thaw cycles, the cell lysate was treated with benzonase nuclease (Merck, Darmstadt, Germany) at 45°C for 15 min and centrifuged two times at 16,000 g for 10 min. The supernatant was used as a virus-containing solution. To measure the titer of the purified virus, the supernatant was dissolved in aCSF. Digital PCR was performed to measure the viral titer using TaqMan MGB probes and the following primer pairs: woodchuck hepatitis virus posttranscriptional regulatory element (WPRE): 5'-VIC-CTGCTTTAATGCCTTTGTAT-MGB-3', forward: 5'-TGCTCCTTTTACGCTATGTGGATA-3', reverse: 5'-CATAAAGAGACAGCAACCAGGATTT-3'; human growth hormone polyA: 5'-FAM-CACAATCTTGGCTCACTG-MGB-3', forward: 5'-GGGTCTATTGGGAACCAAGCT-3', reverse: 5'-GGCTGAGGCAGGAGAATCG-3'. The AAV vector was stored at -80°C in small aliquots until the day of the experiment.

**Immunohistochemistry**

Mice were deeply anesthetized with Avertin and transcardially perfused with heparinized saline (20 U/mL) followed by 4% paraformaldehyde in 0.1 M phosphate buffer (pH 7.4). Brains were removed, post-fixed in 4% paraformaldehyde solution overnight, and transferred to 30% sucrose solution in 0.1 M phosphate buffer (PB) until they sank. A series of 40-μm-thick sections were obtained using a cryostat (CryoStar NX70; Thermo Fisher Scientific, Waltham, MA). For staining, coronal brain sections were immersed in a blocking buffer (10% goat or donkey serum and 0.3% Triton-X in 0.1 M PB) and then incubated with primary antibodies at 4°C overnight. The sections were washed with blocking buffer and incubated with secondary antibodies for 1 hour at room temperature. Brain sections were mounted and examined under a fluorescence microscope (IX73; Olympus, Tokyo, Japan). Primary antibodies and secondary antibodies were diluted in the blocking buffer as follows: anti-Iba1 (RRID: AB_839504, #019-19741, FUJIFILM Wako Pure Chemical Corporation, Osaka, Japan) at 1:2000, anti-calbindin (RRID: AB_10000347, code 300, Swant, Marly, Switzerland) at 1:2000 , anti-GFAP (RRID: AB_477035, G9269, Sigma-Aldrich, St. Louis, MO) at 1:100, anti-GFP (RRID: AB_591819, Code 598, Medical & Biological Laboratories, Tokyo, Japan) at 1:1000, Alexa Fluor 488 donkey anti-rabbit IgG (RRID: AB_2535792, A-21206, Thermo Fisher Scientific) at 1:1000, and Alexa Fluor 647 donkey anti-rabbit IgG (RRID: AB_2752244, ab150075, Abcam, Cambridge, UK) at 1:1000. ProLong Glass Antifade Mountant with NucBlue Stain was used to visualize the nuclei (P36981, Thermo Fisher Scientific). GFP antibody was used to enhance the fluorescence signal of Venus in **Figure 1D**. In this study, GFP, mRFP1 and tdTomato were observed without immunostaining.

**Cell count analysis**

In **Figures 1F and S1**, we manually counted fluorescence-positive cells using ImageJ software (National Institutes of Health, Bethesda, MD). We observed one of every four coronal brain slices and selected three brain slices from each mouse for analysis. We captured multiple fluorescence images in the crus of the cerebellum: blue (NucBlue), green (GFP), red (tdTomato), and infrared (Iba1). Briefly, we analyzed 120 images from 30 regions of 15 brain slices from five mice in **Figure 1F**. In **Figure S1**, we captured multiple fluorescence images: blue (NucBlue), green (GFP), red (tdTomato) in the crus of the cerebellum and analyzed 54 images from 18 regions of nine brain slices from three mice (one week after injection or two days after injection). We randomized the brain slice identifiers for cell counting under blind conditions. We used the GFP signal to confirm the ipsilateral side of the injection. The lengths of the cell-counted areas in the PCL were measured and used to calculate the line density of the tdTomato-expressing cells **(Figure S1)**. The line density of tdTomato-expressing cells in the PCL was used as a normalized index for OXTR expression.

**Fluorescence intensity analysis**

In **Figure S2**, we measured fluorescence intensity of Venus using ImageJ. We observed one of every four coronal brain slices and selected three brain slices from each mouse for analysis. We captured multiple fluorescence images: blue (NucBlue), green (Venus) and red (mRFP1) in the crus of the cerebellum. Briefly, we analyzed 54 images from 18 regions of nine brain slices from three mice. We randomized the brain slice identifiers for cell counting under blind conditions. We used the mRFP1 signal to confirm the ipsilateral side of the injection. To calculate calibrated fluorescence intensity, we subtracted the mean intensity of the neighboring GCL area from the mean intensity of a manually selected area including the PCL and ML.

**Statistics and reproducibility**

Statistical analyses were performed using GraphPad Prism 9 for Windows (GraphPad Software, San Diego, CA). Simple comparisons of the means and SEM in **Figure 1F, S1, and S2** were performed using paired t-test. A P value of less than 0.05 was considered significant in these analyses.

**Figure S1**


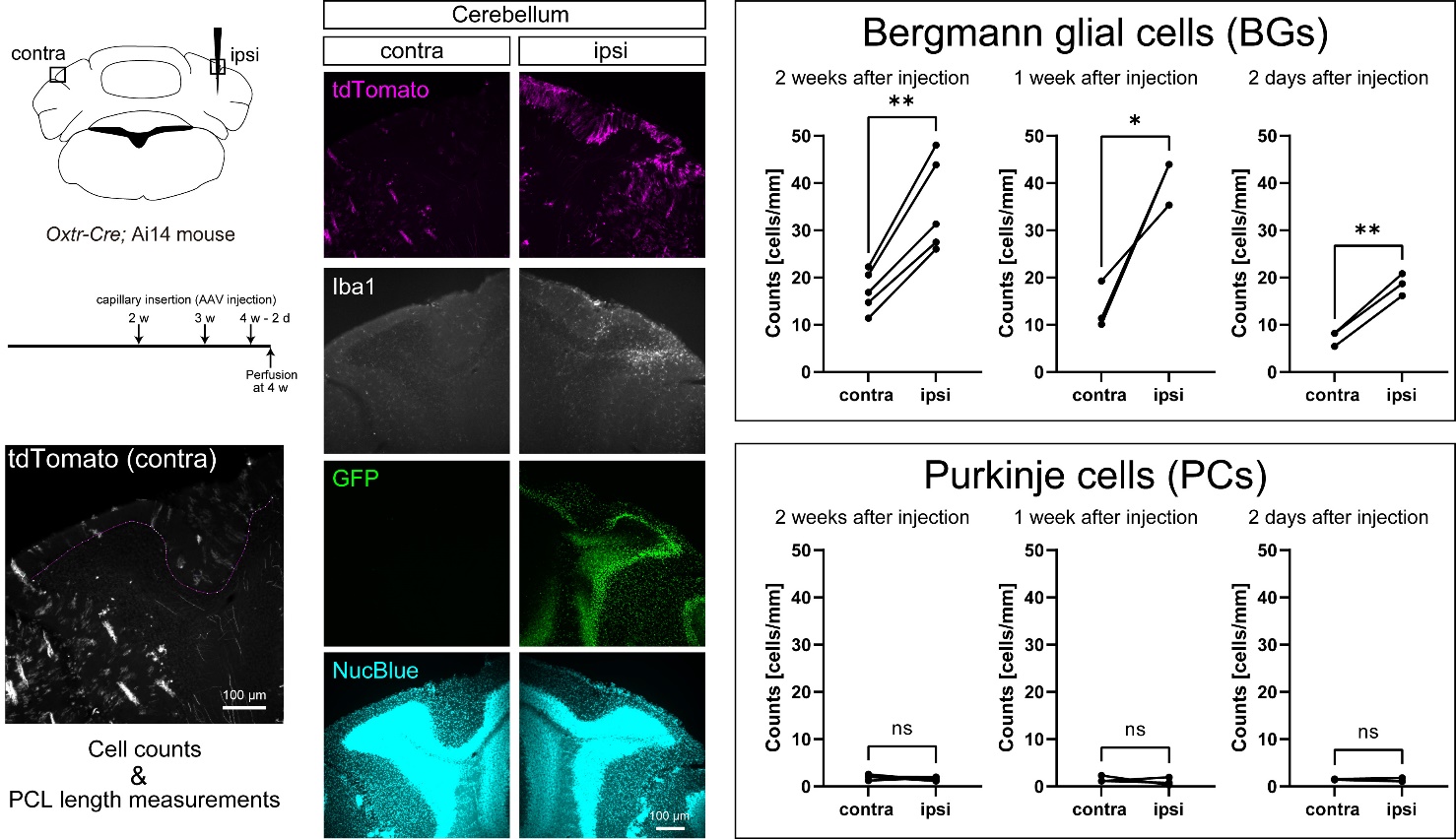


Selective local up-regulation of OXTR in the cerebellum at the site of capillary insertion in 4-week-old *Oxtr-Cre*; Ai14 mice two days, one week, and two weeks after injection. These images were used for analysis in **Figure 1F**. Scale bar = 100 μm. ** P < 0.01 (n = 5 and n = 3) and * P < 0.05 (n = 3). The data of 2 weeks after injection are the same data used in **Figure 1F**.

**Figure S2**


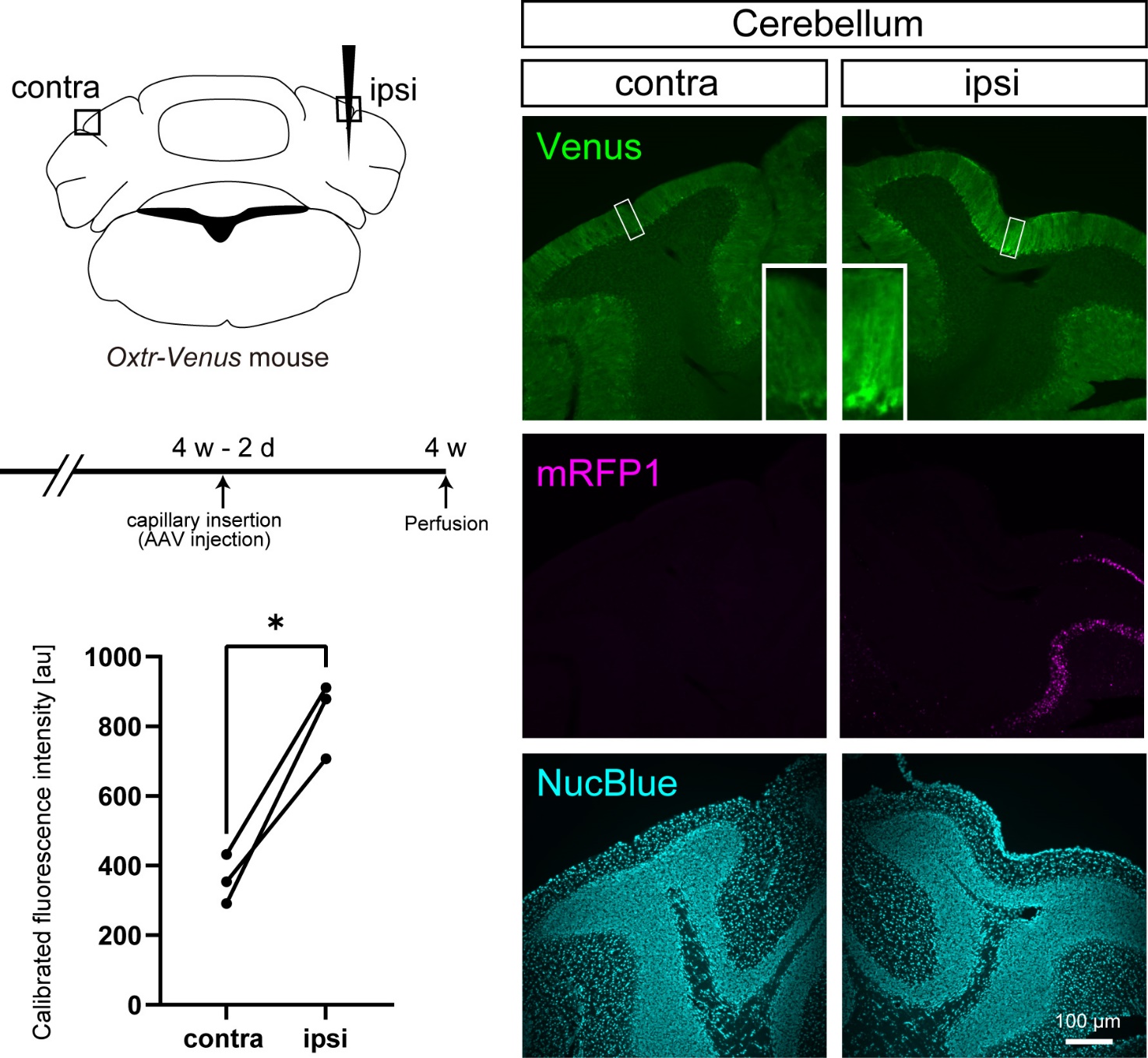


Local upregulation of Venus expression in Bergmann glia of the injured side of the cerebellum in *Oxtr-Venus* mice. A glass capillary was inserted in the right crus of the cerebellum (from lambda -2.6 mm, lateral 2.1 mm, ventral -1.0 mm) of 4-week-old female transgenic mice and 1.2 μL of AAV-CAG-H2B-mRFP1-WPRE (1 × 10 ^12^ vg/mL) was injected. The mice were perfused two days after injection. Scale bar = 100 μm. * P < 0.05 (n = 3).

**Figure S3**


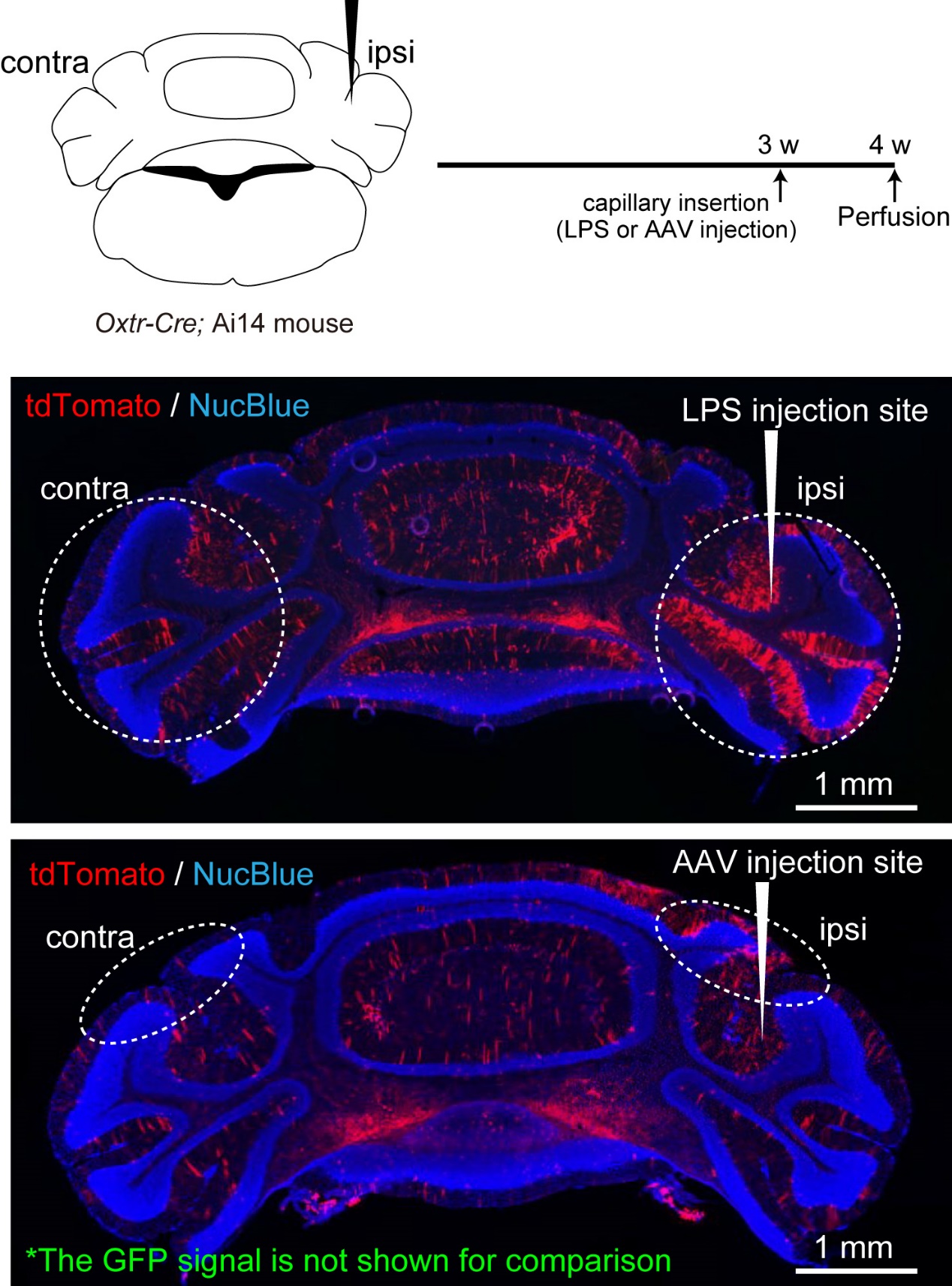


Lipopolysaccharide (LPS) injection induced wide-spread upregulation of OXTR in the deep area of the cerebellum. A glass capillary was injected in the right crus of the cerebellum (from lambda -2.6 mm, lateral 2.1 mm, ventral -1.0 mm) of 3-week-old female double-transgenic mice and 5 μL of LPS (1mg/mL in aCSF) or 1.2 μL of AAV-CAG-H2B-GFP-WPRE (2 × 10 ^12^ vg/mL) was injected. The mice were perfused one week after injection. Scale bar = 1 mm.

**Figure S4**


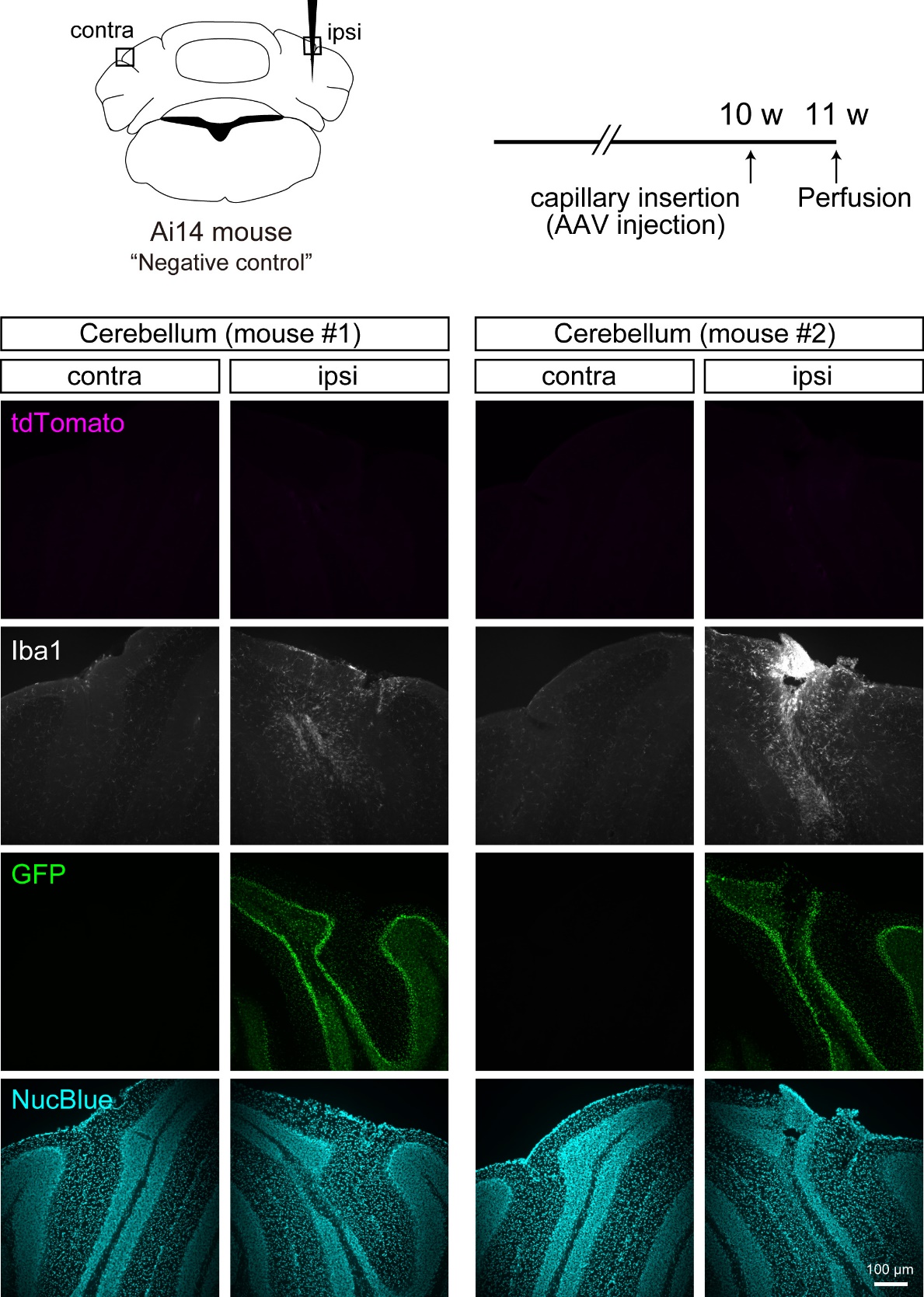


Denial of Cre-independent expression of tdTomato in Ai14 mice. A glass capillary was inserted in the right crus of the cerebellum (from lambda -2.6 mm, lateral 2.1 mm, ventral -1.0 mm) of 10-week-old female transgenic mice and 1.2 μL of AAV-CAG-H2B-GFP-WPRE (2 × 10^12^ vg/mL) was injected. The mice were perfused 1 week after injection. Scale bar = 100 μm.

**Figure S5**


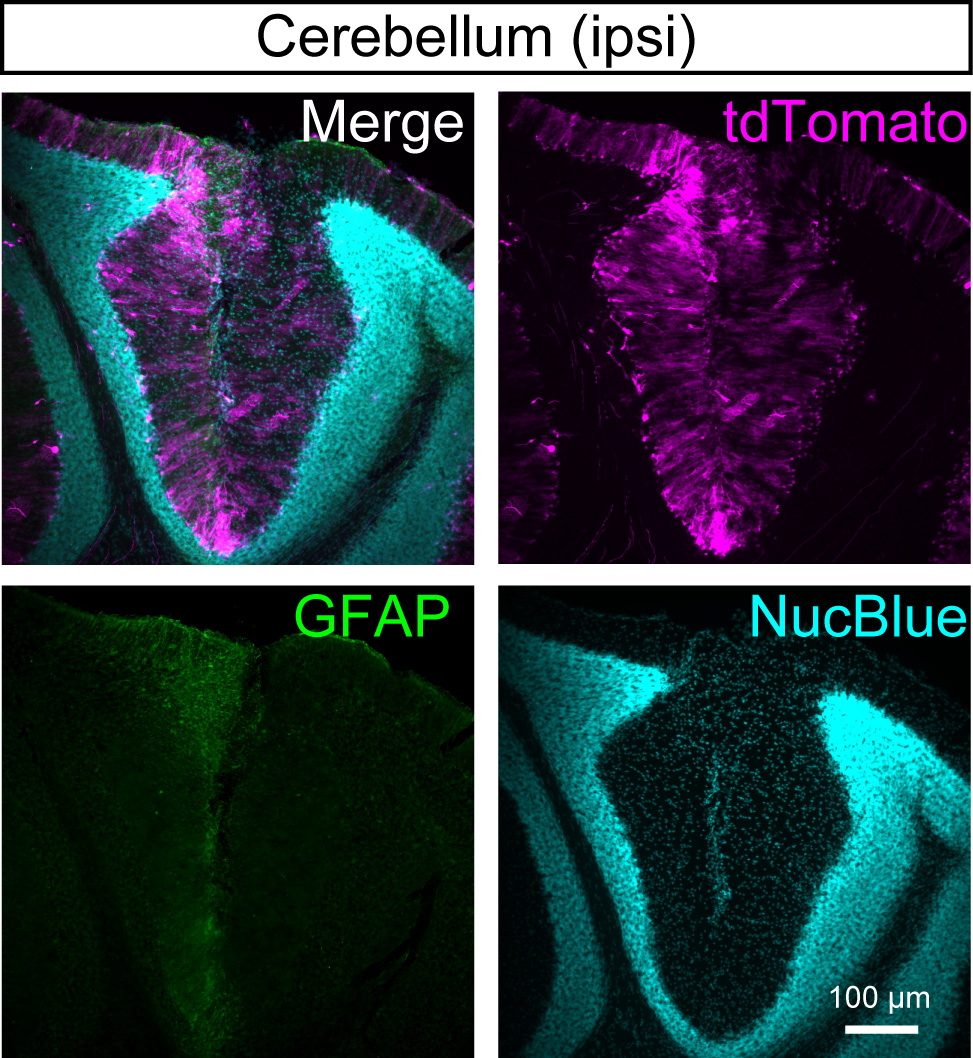


Local upregulation of GFAP expression in Bergmann glia of the injured side of the cerebellum in *Oxtr-Cre*; Ai14 mice. A glass capillary was inserted in the right crus of the cerebellum (from lambda -2.6 mm, lateral 2.1 mm, ventral -1.0 mm) of 7-week-old male double-transgenic mice. The mice were perfused 1 week after injection. Scale bar = 100 μm.
