# Supplementary figures and images for: Cerebellar damage with inflammation upregulates oxytocin receptor expression in Bergmann Glia
Source: Mol Brain. 2024 Jun 28;17:41. doi: 10.1186/s13041-024-01114-5 (PMC11214225; doi:10.1186/s13041-024-01114-5)

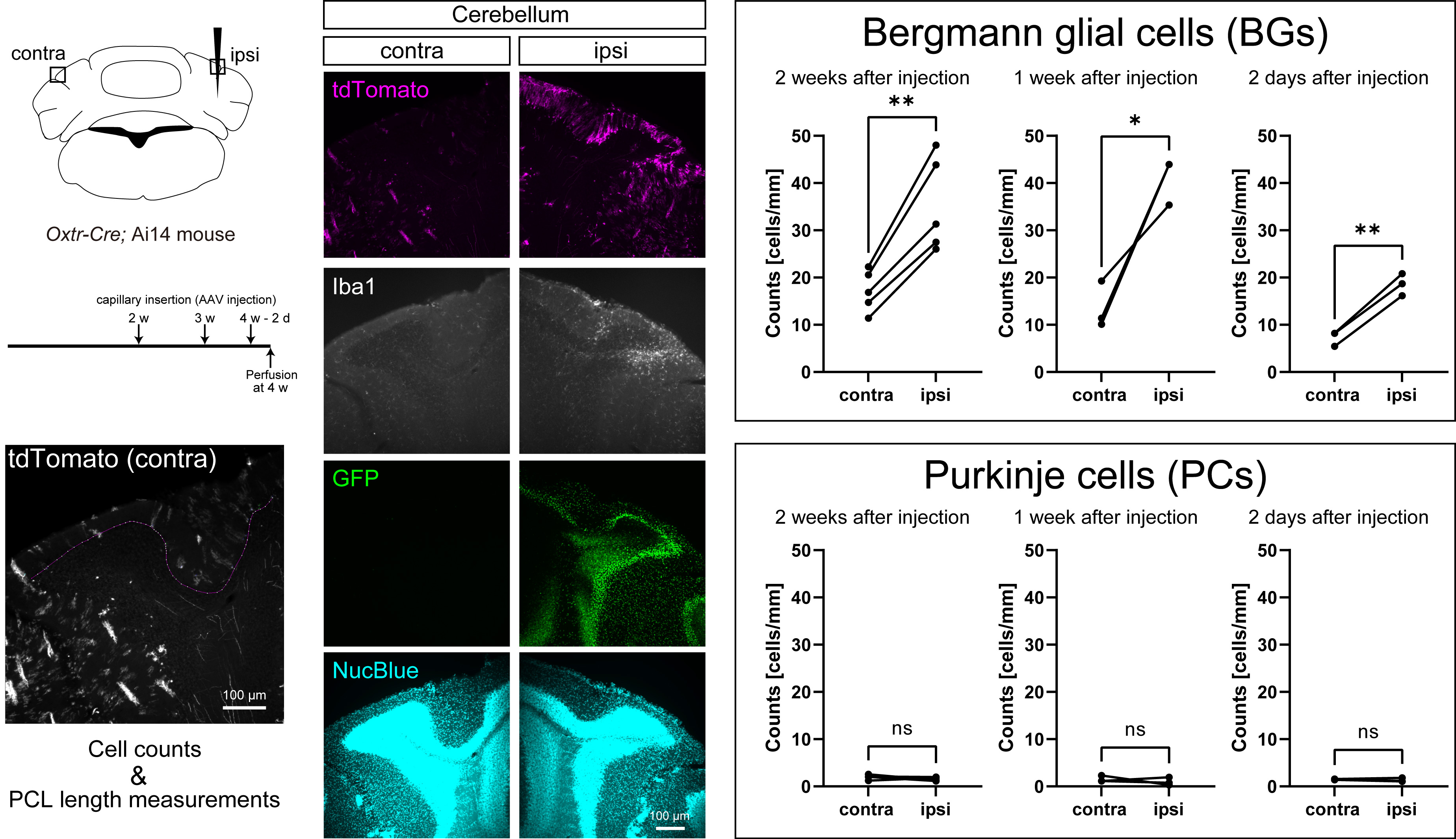

Supplement: Supplementary file 2 — Supplementary Material 2 [file 13041_2024_1114_MOESM2_ESM.jpg]

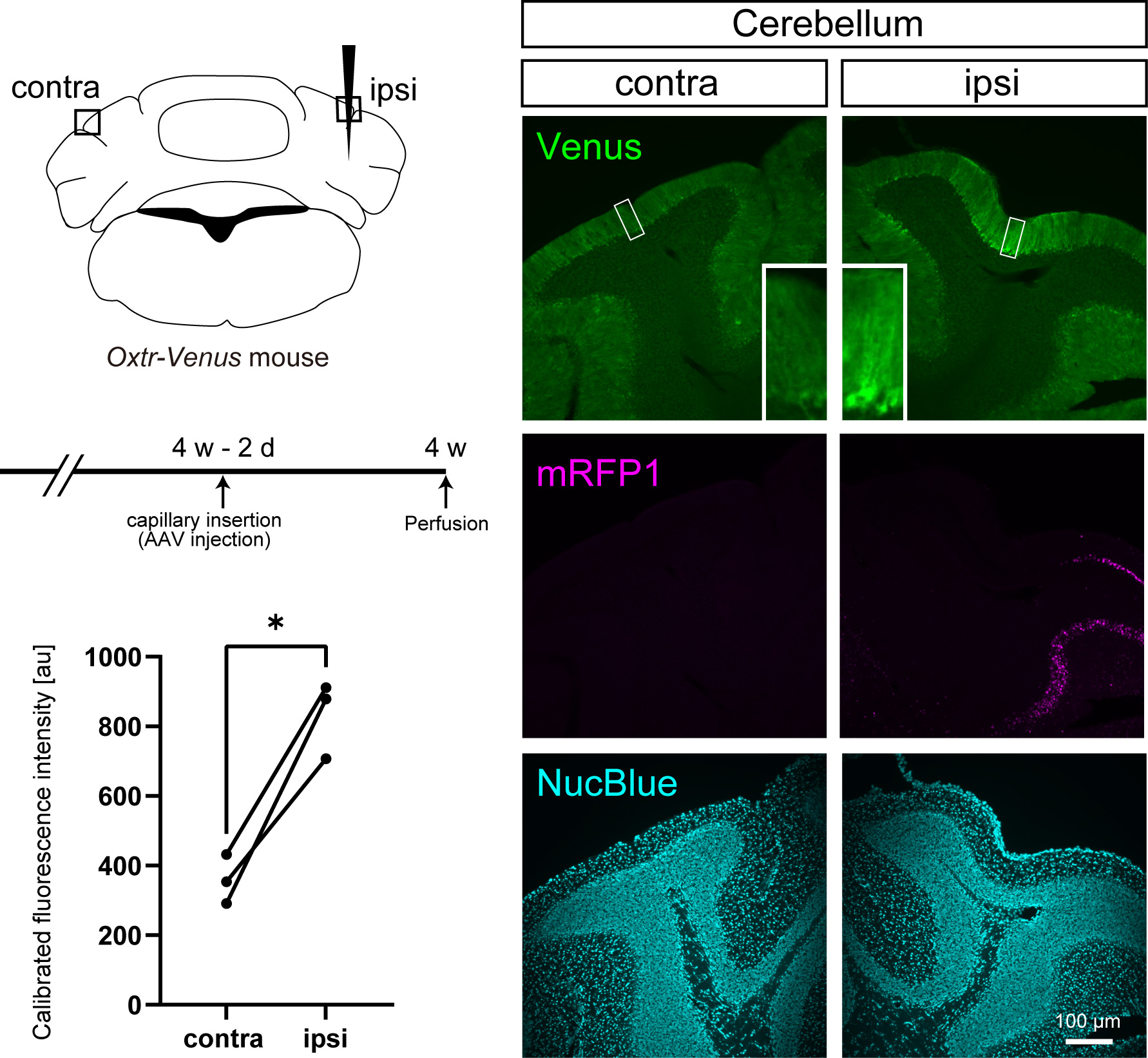

Supplement: Supplementary file 3 — Supplementary Material 3 [file 13041_2024_1114_MOESM3_ESM.jpg]

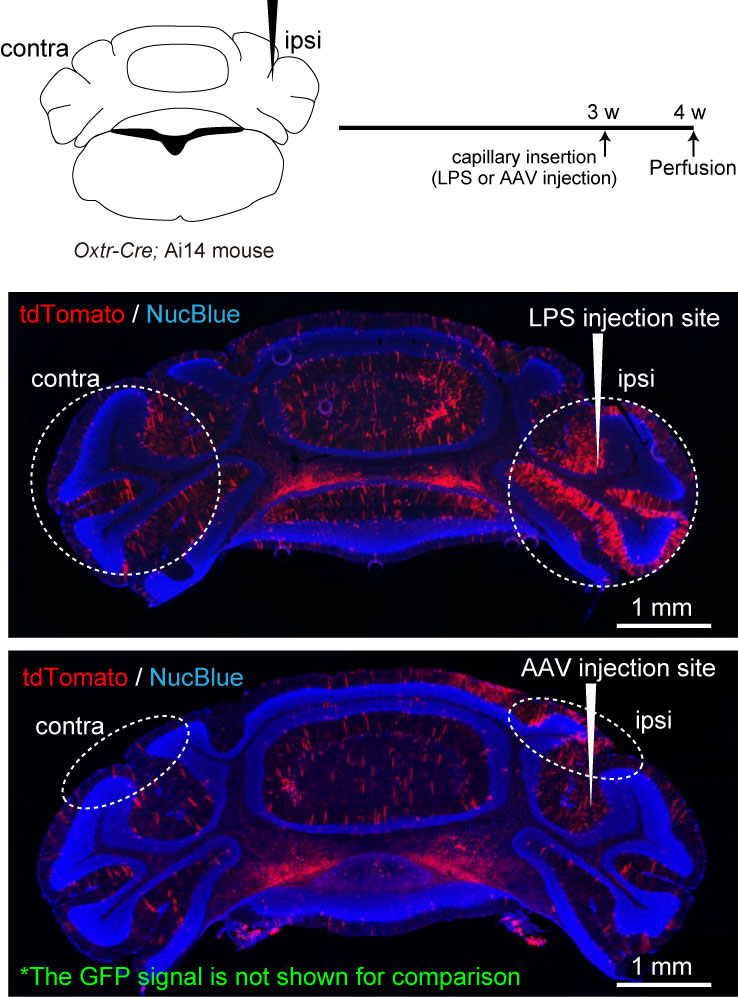

Supplement: Supplementary file 4 — Supplementary Material 4 [file 13041_2024_1114_MOESM4_ESM.jpg]

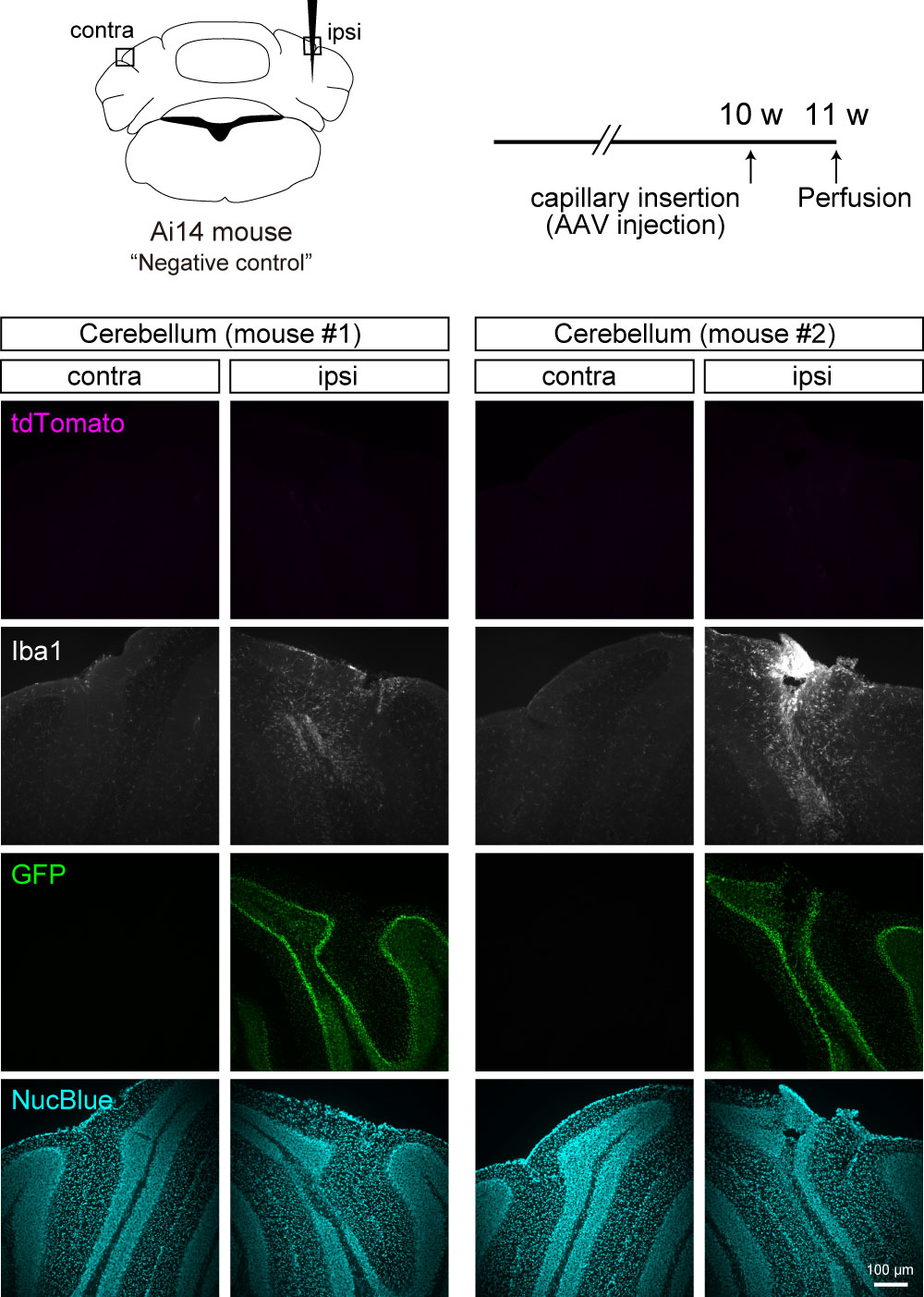

Supplement: Supplementary file 5 — Supplementary Material 5 [file 13041_2024_1114_MOESM5_ESM.jpg]

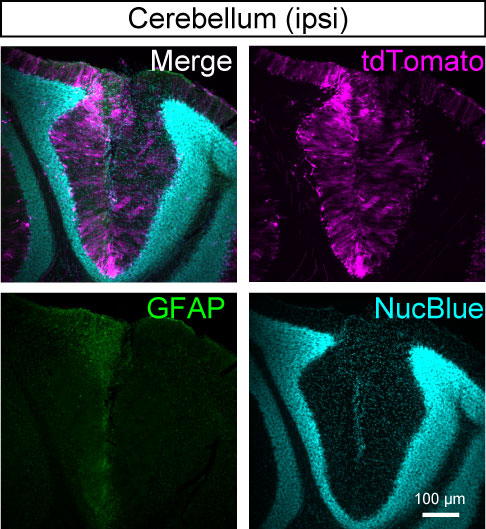

Supplement: Supplementary file 6 — Supplementary Material 6 [file 13041_2024_1114_MOESM6_ESM.jpg]
